# Bond strength and interfacial analysis of six CoCr alloys made by conventional casting and selective laser melting

**DOI:** 10.1111/jopr.13918

**Published:** 2024-08-26

**Authors:** Youssef S. Al Jabbari, Konstantinos Dimitriadis, Aref Sufyan, Spiros Zinelis

**Affiliations:** ^1^ Dental Biomaterials Research and Development Chair and Prosthetic Dental Sciences Department College of Dentistry King Saud University Riyadh Saudi Arabia; ^2^ Department of Biomaterials School of Dentistry National and Kapodistrian University of Athens Athens Greece; ^3^ Dental Biomaterials Research and Development Chair College of Dentistry King Saud University Riyadh Saudi Arabia; ^4^ Department of Biomaterials School of Dentistry National and Kapodistrian University of Athens, Athens, Greece and Dental Biomaterials Research and Development Chair King Saud University Riyadh Saudi Arabia

**Keywords:** 3‐point bending, CoCr alloys, ISO 9693, metallo‐ceramic bond strength, PFM alloys, SEM/EDX

## Abstract

**Purpose:**

To investigate the effects of the elemental composition and the manufacturing process of cobalt chromium–molybdenum (CoCr–Mo), cobalt chromium–tungsten (CoCr–W), and CoCr–Mo‐W alloys on metal–ceramic bond strength.

**Materials and Methods:**

Six CoCr‐based alloys were included in this study, a were classified into three different groups depending on their elemental composition (*Ν* = 10, for each group). The first group had molybdenum (Mo) as the third alloying element, the second group contained tungsten (W) (without Mo), and the third group included both alloying elements. The groups were further divided by the manufacturing process (casting or selective laser melting, SLM). Interfacial analysis was carried out using backscattered electron imaging (BEI) and energy‐dispersive X‐ray microanalysis (EDX) operating in line scan mode. The metal‐ceramic bond strength was tested by a 3‐point bending test according to the ISO 9693 requirements. The fracture mode of all specimens was examined under a stereomicroscope. The bond strength results were statistically analyzed by 2‐way ANOVA and Tukey's multiple comparison post hoc test (*a* = 0.05).

**Results:**

A continuous interface with the porcelain was found without pores, debonding areas, or other defects. Of the major elements found at the interface, Co showed the highest diffusion rate, while titanium (Ti) had the lowest diffusion rate. No statistically significant differences were identified in metal‐ceramic bond strength either among materials or between manufacturing processes. The fracture mode was found to be cohesive for all specimens.

**Conclusions:**

The metal‐ceramic bond strength is independent of the current CoCr alloy type and manufacturing process when comparing conventional casting and SLM. Interfacial analysis revealed no differences between the tested groups.

In recent decades, a cobalt chromium (CoCr) alloy has been used in dentistry for the production of porcelain‐fused‐to‐metal (PFM) prosthetic restorations due to its acceptable mechanical properties, corrosion resistance, and good biocompatibility.^1^, ^2^, ^3^ While these restorations mainly contain Co and Cr, other alloying elements such as powerful strengthening agents tungsten (W) and molybdenum (Mo) or elements used to increase fluidity, modify thermal expansion, control oxidation and refine grain size (manganese(Mn), silicon (Si), iron (Fe), niobium (Nb) and gallium (Ga)) are also included in the composition of these alloys.^3^, ^4^, ^5^ Considering that (i) ISO 9693^6^ does not contain any restrictions on the elemental composition of alloys for metal–ceramic systems,^4^ in recent decades, manufacturers have developed compositions using mainly W instead of or in combination with Mo as the alloying element of the CoCr dental alloy. Currently, there are three types of CoCr alloys for the fabrication of PFM restorations, classified as CoCr–Mo, CoCr–W, and CoCr–Mo–W, whose formulations are dominated by Co, Cr, (Mo or W or both of them) alloy types and traces of the remaining elements.

In the metallurgy of CoCr alloys, Mo and W play similar roles, as both improve mechanical strength due to solid solution strengthening and carbide formation and also enhance corrosion resistance. Although Mo was initially used extensively instead of W, the development of CoCr–W alloys has evolved in recent decades because the addition of W increases the volume fraction of the face centered cubic (FCC) phase at room temperature.^7^, ^8^ This phase has a positive effect on yield strength and fatigue, and the capacity to absorb energy due to the transformation of FCC to hexagonal closed packed structure (HCP).^9^ In contrast to its beneficial effects, the use of W leads to the simultaneous precipitation of the brittle sigma phase (i.e., an undesirable brittle intermetallic compound).^7^, ^8^ In any case, the presence of different phases or elements strongly affects the nature of native oxides at the alloy surface and is therefore of great importance for the oxidation stage of dental alloys at high temperatures^10^ and consequently for the metal‐ceramic bond.^7^

In dental laboratories, there has been a rapid shift in manufacturing dental metallic prostheses from the lost‐wax casting process to the computer‐aided design and computer‐aided manufacturing (CAD–CAM) process, which includes both subtractive (soft or hard milling) and additive (selective laser melting, SLM) manufacturing technologies.^11^ These changes in manufacturing processes have led to changes in physical properties, such as microstructure, resulting in the differentiation of mechanical and electrochemical properties and their biocompatibility compared to conventional casting.^2^, ^12^, ^13^, ^14^, ^15^ In the fabrication of PFM restorations, the abovementioned CAD–CAM processes have been directly adopted. However, since CoCr alloys fabricated by different manufacturing processes do not share similar microstructures,^14^ the nature of the oxidation surface can change with consequences to the metal‐ceramic bond strength.^16^

As the primary requirement for the longevity and success of restorations, the bond strength between various dental porcelains and CoCr alloys prepared by casting or SLM has been characterized in many previous reports.^17^, ^18^, ^19^, ^20^, ^21^, ^22^, ^23^, ^24^, ^25^, ^26^, ^27^, ^28^, ^29^, ^30^, ^31^ Surprisingly, all reports deal with the CoCr–Mo–W alloy system, except for one report on the CoCr–W alloy system,^23^ which found significant differences between the cast and SLM groups. In contrast, no statistically significant differences were found in all studies for the CoCr–Mo–W system^18^, ^19^, ^20^, ^21^, ^22^, ^24^, ^25^, ^26^, ^27^, ^28^, ^29^, ^30^ (Figure 1). Although most of the studies used the same dental porcelain (Vita VMK Master) and the 3‐point bending test, as described by ISO 9693, the bond strength values ranged from 32 to 75 MPa and thus cannot be considered conclusive. Summarizing the current knowledge, the CoCr–Mo system has been completely omitted from the analysis, and the main hypothesis cannot yet be answered as to whether the material or the manufacturing process actually have an effect on the metal‐ceramic bond strength.

**FIGURE 1:**
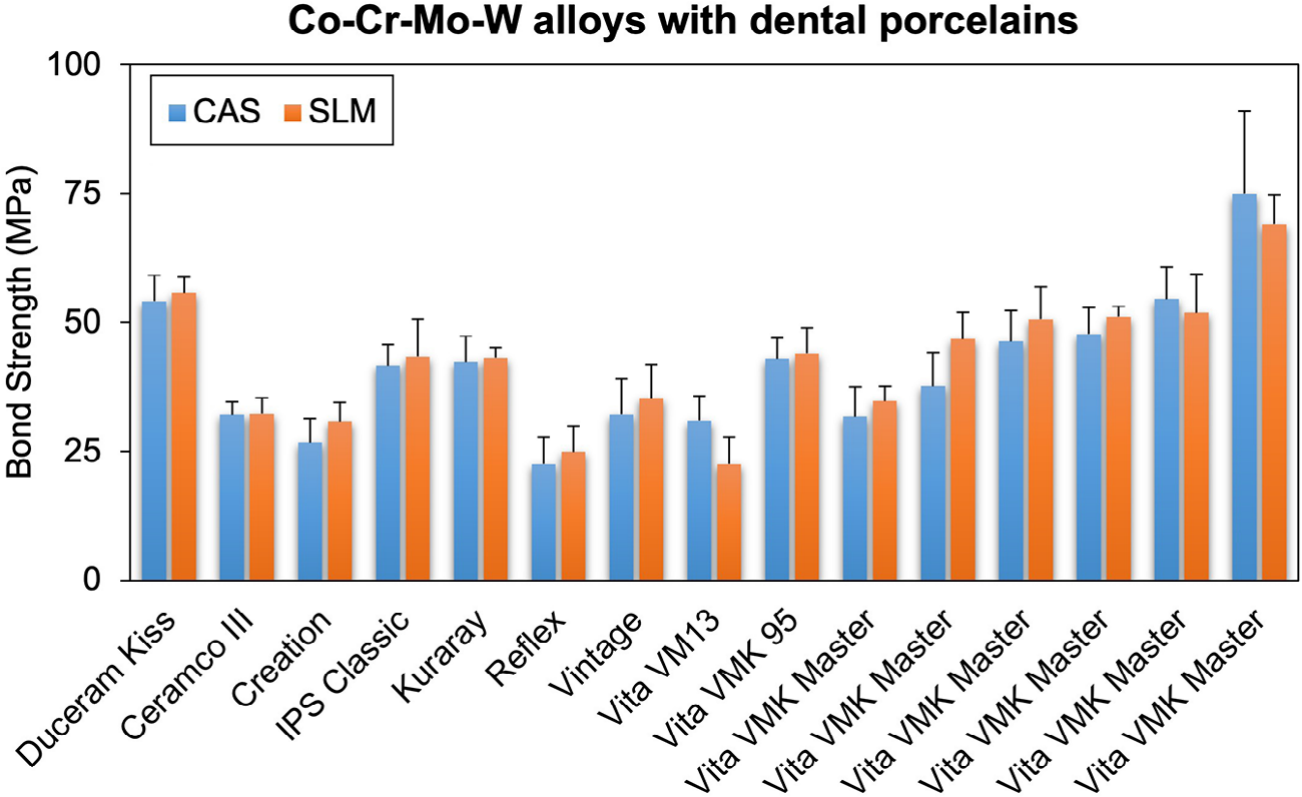
Literature data for the bond strength of various dental porcelains to CoCr alloys belongs to the CoCr*–*Mo*–*W system. No statistically significant differences were found in all studies between groups prepared by conventional casting and SLM technology.^18^, ^19^, ^20^, ^21^, ^22^, ^24^, ^25^, ^26^, ^27^, ^28^, ^29^, ^30^ All data were acquired according to ISO 9693 specifications.

Therefore, the objective of the present work was to investigate the effects of the elemental composition and the manufacturing process of CoCr–Mo, CoCr–W, and CoCr–Mo–W alloys on the metal‐ceramic bond strength according to ISO 9693. The null hypothesis was that no significant differences would be found either among different alloy types or between manufacturing processes.

## MATERIALS AND METHODS

### Specimen preparation

Six porcelain fused to metal (PFM) CoCr‐based alloys indicated for manufacturing were included in this study. The alloys were initially classified into three groups based on the third major alloying element. The first group had Mo without W, the second W without Mo, and the third had both elements. Each group was divided into two subgroups based on manufacturing process (traditional casting, and selective laser melting SLM). Brand names, elemental composition, manufacturing process, thermal expansion coefficient, and code of alloys used in this study are listed in Table 1.

**TABLE 1:** Brand names, elemental composition (as provided by the manufacturers), manufacturing method, thermal expansion coefficient (TEC), and codes of all materials tested.

	V‐Comp^a^	ST2724G^b^	Remanium Star CL^c^	Remanium Star CL^c^	Wirobond 280^d^	EOS‐CobaltChrome SP2^e^
Co	61.1 ± 2	62.5 (min)	60.5	60.5	60.2	61.8–65.8
Cr	32 ± 2	29	28	28	25	23.7–25.7
Mo	5.5 ± 1	5.5			4.8	4.6–5.6
W			9	9	6.2	4.9–5.9
Si	<1	<1	1.5	1.5	<1	0.8–1.2
Mn	<1	<1		<1	<1	<0.1
Fe		<1				<0.5
Nb				<1		
Ga					2.9	
N				<1		
Manufacturing process	Casting	SLM	Casting	SLM	Casting	SLM
TEC 10^−6^ K^−1^ (25°C–500°C)	14.25	14.5	14.1	14.1	14.0	14∼14.5
Code	Mo‐C	Mo‐L	W‐C	W‐L	MoW‐C	MoW‐L

^a^ Dentsply, Elephant Dental, Hoorn, The Netherlands.

^b^ SINT‐TECH, Riom, France.

^c^ Dentaurum, Inspringen, Germany.

^d^ Bego, Bremen, Germany.

^e^ EOS, Munchen, Germany.

For each group, 11 rectangular specimens with final dimensions 25 × 3 × 0.45∼0.55 mm were prepared. The specimens of casting groups (Mo–C, W–C, and MoW–C) were manufactured by the traditional lost wax casting procedure employing a centrifugal casting machine (Ducatron S3, Ugin'Dentaire, Seyssins, France) and induction melting according to their manufacturers’ instructions. Wax patterns (IQ sticks, Yeti Dental, Engen, Germany) measuring approximately 26 × 3.5 × 0.8 mm were invested with a phosphate‐bonded silica‐based investment material (GC Stellavest, GC Europe, Leuven, Belgium).

The specimens of SLM groups (Mo–L, W–L, and MoW–L) were prepared using the SLM devices PM 100 (Dental System, Phenix Systems), Mlab (ConceptLaser), and EOS Laser Sintering M270, (EOS), respectively. A thorough description of the manufacturing process and materials used can be found elsewhere.^4^ Then the specimens were ground on all sides up to 1200 grit SiC paper under continuous water cooling and 300 rpm rotation rate up to final dimensions (25 mm × 3 mm × 0.45−0.55 mm) in a grinding polishing machine (Dap V, Struers, Bellarup, Denmark).

### Modulus of elasticity

Two rectangular specimens from each group, as required by ISO 9693,^6^ were subjected to a 3‐point bending test to determine the modulus of elasticity employing a universal testing machine (Tensometer10, Monsanto, Swindon, UK). The 3‐point bending apparatus was made from hardened steel with a span between supports of 20 ± 0.1 mm. Both supports and loading piston had smooth surfaces with a radius of 1.0 ± 0.1 mm. The specimens were loaded at the center with a crosshead speed of 1.5 mm/min, and the force and deflection data were recorded. The bending elastic modulus (*E_b_*) was calculated according to the following formula:

(1) $$E_{b}=\frac{L^{3}\Delta P}{bh^{3}\Delta d}\;$$

*L* is the distance between supporting rods (20 mm), *b* is the specimen width (3 mm), *h* is the specimen thickness (0.5 mm), and Δ*P* and Δ*d* are the load and deflection increment, respectively, between two selected points in the elastic portion of the curve. The mean value of the modulus of elasticity was used to determine the k constant as required by ISO 9693.

### Evaluation of bond strength according to ISO 9693

Six specimens from each group were ground from one side up to 2000 grit and polished with 9, 3, and 1 μm diamond pastes (DP Paste, Struers). Before applying the porcelain, a final cleaning was performed with hot distilled water in an ultrasonic cleaner for 10 min in 95% ethanol. The polished surface was sandblasted with Al_2_O_3_ particles, and successive layers of porcelain (bonding agent, two opaques, two dentin and glaze) were applied at the center of each specimen over an 8 mm length and 1 mm thickness according to the ISO 9693^6^ requirements. The porcelain used was the initial MC with CTE 13.3 (25°C–500°C; *10^−6^ K^−1^). Table 2 shows the firing parameters for each layer. A dental porcelain furnace (Multimat Touch, Dentsply DeTrey, Konstanz, Germany) was used to fabricate the metal–ceramic specimens.

**TABLE 2:** Firing schedules of the veneering procedure for GC initial MC porcelain (according to the manufacturer's instructions).

Product name	Preheating temp. (°C)	Drying time (min)	Heating rate (°C/min)	Vacuum	Final temp. (°C)	Holding time (min)
INmetalBond	550	6	80	Yes	980	1
Opaque	550	6	80	Yes	940	1
Dentin	580	6	55	Yes	900	1
Glaze	480	2	45	No	850	1

According to ISO standard 9693, a 3‐point bonding test was applied to evaluate the metal‐porcelain bonding strength until debonding of the porcelain layer in a universal testing machine with a speed of 1.5 mm/min. Porcelain debonding was recognized by a sudden decrease in the force–deflection plot, and the debonding load was recorded for each specimen. The calculation of bond strength was determined by the formula provided by ISO 9693:^6^

(2) $$\mathrm{Bond}\;\mathrm{strength}\left(\mathrm{MPa}\right)=k*F$$

where *k* is a coefficient derived from the ISO diagram based on the bending modulus of elasticity and *F* stands for the debonding load in *N*.

### Fracture mode

The fracture mode of all fractured specimens was examined under a stereomicroscope (Leica M80, Leica Microsystems, Wetzlar, Germany). The fractures are characterized as adhesive (fracture at the interface), cohesive (fracture within the material structure), and mixed (sites with adhesive and cohesive failure). Photographs of metallic substrates and overlayed porcelain were acquired under incident light at 5 and 10× nominal magnification using a digital camera (Leica DFC295, Leica Microsystems) coupled to the microscope.

### Interfacial characterization

Three specimens from each group were ground from one side with SiC paper of up to 2000 grit, polished with 9, 3, and 1 μm diamond pastes (DP Paste, Struers) and cleaned in an ultrasonic bath for 10 min in 95% ethanol. Then, the specimens were covered with porcelain directly on the polished surfaces, omitting the step of sandblasting with Al_2_O_3_ contrary to the manufacturers’ instructions following the procedure described above. Subsequently, the specimens were embedded in resin, ground with SiC paper of up to 2000 grit, and polished with 6, 3, and 1 μm diamond pastes (DP Paste, Struers). Then, the specimens were sputter‐coated with carbon in a sputter‐coating unit (SCD 004 Sputter‐Coater with OCD 30 attachment, Bal‐Tec, Vaduz, Liechtenstein). The metal–ceramic interface was examined with scanning electron microscopy (SEM, Quanta 200, FEI, Hillsboro, OR, USA). The interfaces were imaged with backscattered electron imaging (BEI) employing a solid‐state backscattered detector under a 20 kV acceleration voltage, a 110 μA beam current, and 4000× nominal magnification. Then, the elemental distribution at the interface was recorded across the metal–ceramic interface by energy‐dispersive X‐ray microanalysis (EDX) employing a spectrometer (Quantax, Bruker, Berlin, Germany) attached to the SEM microscope equipped with a slew‐window silicon drift detector (X Flash 6|10, Bruker) under the same accelerating voltage and beam current and a 25,000x nominal magnification. Elemental composition profiles were collected across a 5 μm line acquiring 20 point spectra per μm (100 points, step: 0.05 μm). The results were acquired and quantified by dedicated software (ESPRIT ver. 1.9, Bruker, Berlin, Germany) employing ZAF (atomic number—absorbance—fluorescence) correction routines. The length of elemental compositional alterations at the interface was roughly estimated by the line scans for the major elements of alloy (Co and Cr) and Inmetalbonder (titanium (Ti) and oxygen (O)) by isolating each elemental profile for the sake of clarity.

### Statistical analysis

The results of bond strength were statistically analyzed by 2‐way ANOVA and Tukey's multiple comparison post hoc test employing alloy type and manufacturing process as discriminating variables (*a* = 0.05). Initially, the data were checked for the presence of outliers with the Grubbs test, followed by normality and equal variance by Kolmogorov‒Smirnov and Brown‐Forsythe tests, respectively (*a* = 0.05). The statistical analysis was carried out by OriginPro (OriginLab Corporation, Northampton, MA, USA).

## RESULTS

All specimens debonded at the metal‐ceramic interface at one end of the ceramic layer, as indicated in ISO 9693, and therefore, all specimens were included in the study. The mean values of the bond strength along with the standard deviations are presented in Table 3.

**TABLE 3:** Mean values and standard deviation (in parentheses) of all systems tested. No statistically significant differences were determined either among materials or between manufacturing techniques.

System	Cast	SLM
CoCr‐Mo	50(4)^A1^	49(5)^A1^
CoCr‐W	52(5)^A1^	47(6)^A1^
CoCr‐Mo‐W	49(6)^A1^	51(6)^A1^

Same capital letter denotes no statistically significant differences among different alloy systems (columns) and same number between manufacturing process (rows).

No statistically significant differences (Table 3) were identified either among materials (*p* = 0.942) or between manufacturing processes (*p* = 0.579), and no interactions were found between the independent variables (*p* = 0.380).

**FIGURE 2:**
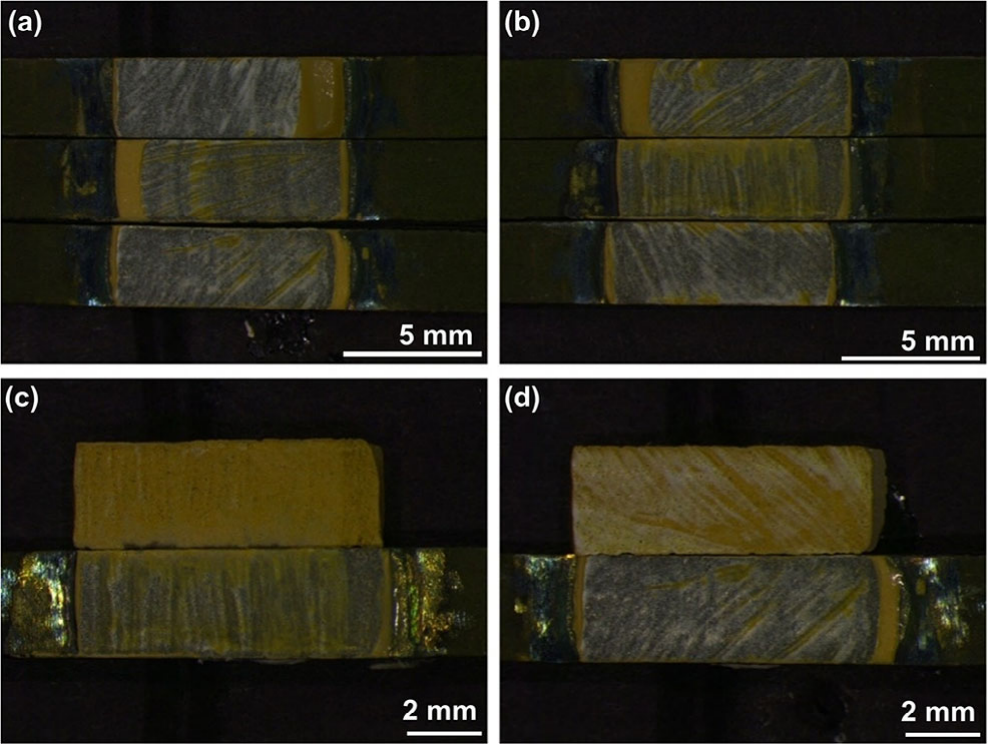
The metallic substrates after debonding (a, b) at 5× nominal magnification. Mutual surfaces of metallic substrate and porcelain at 10× nominal magnification (c,d).

Figure 2 shows representative images from the fracture mode characterization. In Figure 2a and b, the metallic specimens appear covered with white and yellowish layers that are probably attached to the ceramic material, so the fracture should be characterized as cohesive in dental porcelain. Figure 2c and d depict the mutual surfaces of the metallic substrate and porcelain. Given that the white areas are lower than the yellow ones (Figure 2d), they should be attributed to the cohesive fracture of the INmetalBonder, while the yellow color should be attributed to the opaque layer. Therefore, the fracture is mixed as combined adhesive and cohesive fractures within the layers of dental porcelain.

**FIGURE 3:**
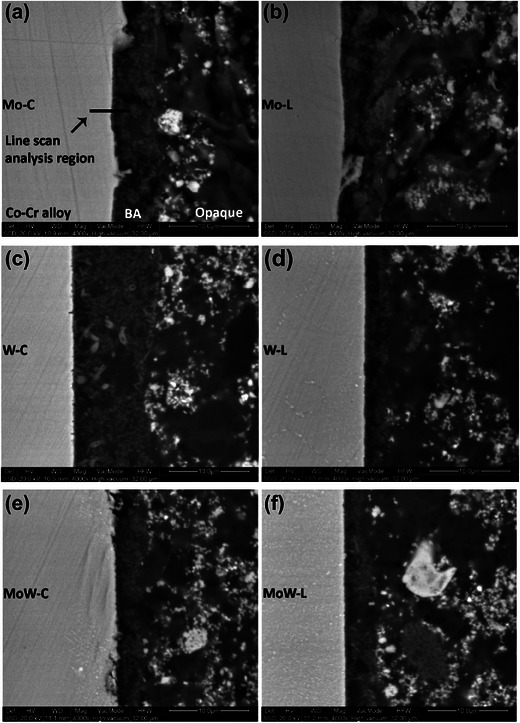
Backscattered electron images of the alloy porcelain interfaces for the materials tested (4000× nominal magnification). The alloy appears on the left, the bonding agent in the middle and the opaque material on the right side of each image.

**FIGURE 4:**
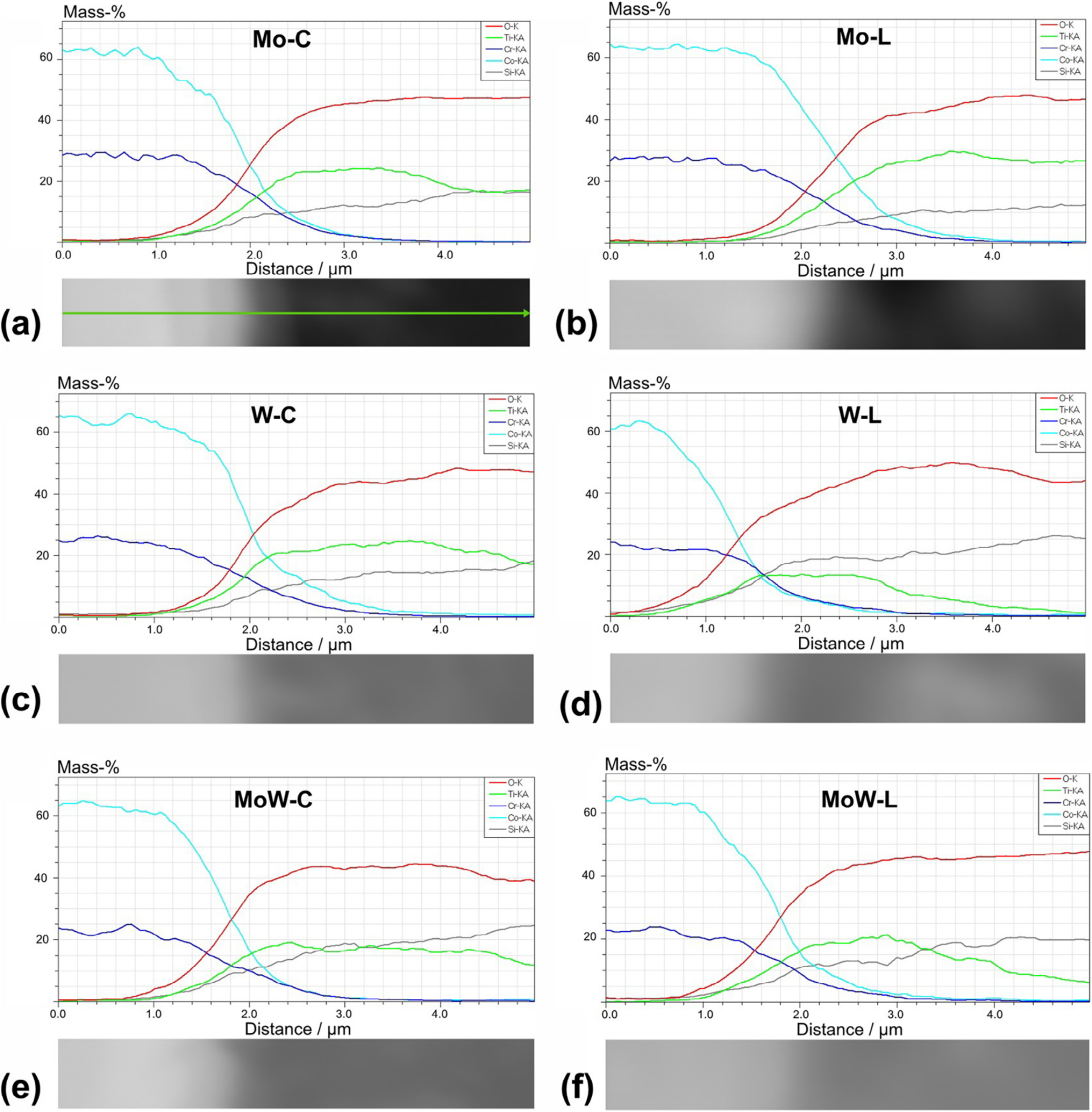
EDX line profile analysis performed across the interface between the alloy and the INmetalbond. All figures share the same coloring for each element. The region where the line scan is recorded is shown with a green horizontal line in (a) but omitted from the remaining figures for the sake of clarity. Full line scan length 5 μm. Nominal magnification 25,000×.

**FIGURE 5:**
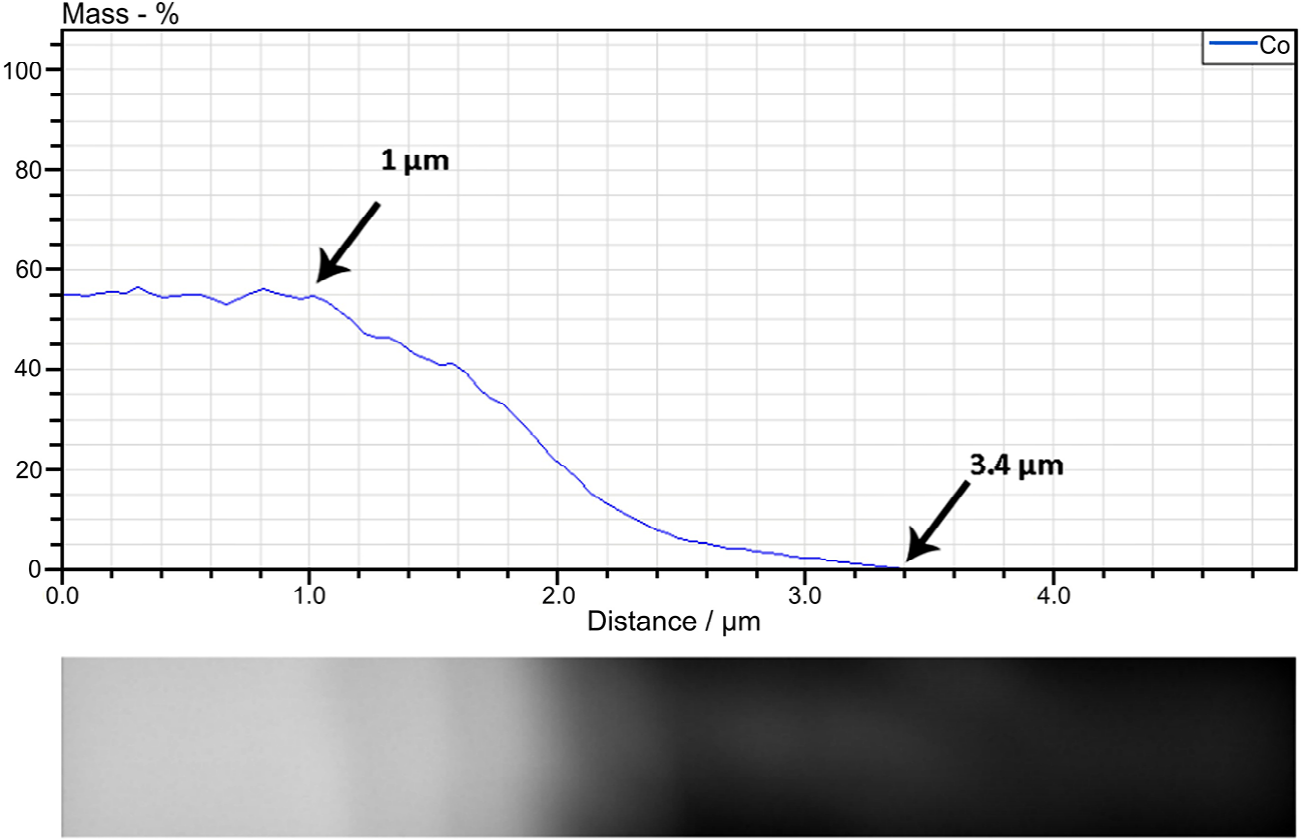
Line scan profile where the locations of the maximum and minimum values were used to estimate the diffusion range of each element at the interface.

Representative BEIs from the interface of all tested materials are depicted in Figure 3. All materials demonstrated a continuous interface between the CoCr alloys and the INmetalbond. The results of the EDX line profile analysis are shown in Figure 4. The probed elements demonstrated a steady decrease (Co, Cr) or increase (Si, O, and Ti) from the alloy toward the INmetalbond. Figure 5 illustrates the line scan of a single element and the location of the maximum and minimum values used to estimate the diffusion range. The results for all elements and groups are summarized in Table 4.

**TABLE 4:** Mean values in microns of four elements indicating their diffusion range at the interface of the porcelain‐alloy systems (*n* = 2).

Group	Co	Cr	Ti	O
Mo*–*C	3.2	2.8	1.9	2.4
Mo*–*L	3.1	2.9	2.1	2.2
W*–*C	3.2	3.1	1.6	2.3
W*–*L	2.8	2.6	2.2	3.0
MoW*–*C	2.6	2.5	1.6	2.1
MoW*–*L	3.1	3.0	1.9	2.1

## DISCUSSION

According to the results of this study, the null hypothesis should be accepted since no significant differences were demonstrated either among different alloy types or between the manufacturing processes.

Only one company provides the same alloy (Remanium, CoCr‐W) as ingots for casting and as powder for SLM, so alloys with as similar a composition as possible should be chosen for the other two categories (Table 1). In addition, it is well known that the differences in CTE between porcelain and alloy lead to residual stresses at the interface, which act as a contributing factor to metal‐ceramic bond strength.^32^, ^33^ To mitigate the effects of this parameter, two measures were taken. First, the selected alloys share CTEs with less than 0.5 10^−6^ K^−1^ and a porcelain with bonding agent was used for all groups. As claimed by the manufacturer, the selected bonding agent does not increase the bond strength but neutralizes the differences in CTEs, allowing a wider span of thermal compatibility between alloys and porcelain.

No significant interaction was found between the independent variables, implying that alloy types and manufacturing processes affect the metal‐ceramic bond strength independently from the state of the other factor (*p* = 0.380). The main acceptance criterion of ISO 9693 (four out of six specimens have bond strength higher than 25 MPa) was fulfilled by all groups, as no specimen showed bond strength lower than 25 MPa. To the best of our knowledge, the selected porcelain has not been tested before, so a comparison with previous data is not possible. The results of our study for CoCr*–*Mo*–*W are in full accordance with previously published data, where no differences were found between groups manufactured by conventional casting and SLM technology.^18^, ^19^, ^20^, ^21^, ^22^, ^24^, ^25^, ^26^, ^27^, ^28^, ^29^, ^30^ From the data in Figure 1, it is clear that the bond strength depends on porcelain but remains independent of the manufacturing process. Regarding the CoCr*–*W system, only one previous study^23^ reported differences in bond strength (casting 38.08 ± 3.82 MPa, SLM 41.24 ± 3.75 MPa), which may be appended to the different porcelain used or to the fact that a modified SLM method was commonly known as the “direct process powder bed system”, where a laser beam hits the surface at different points simultaneously to decrease thermal stresses during 3D building.^34^ The cohesive fracture mode of all groups is in accordance with previous findings when INMetalbonder is used as an intermediate layer between opaque and alloy surfaces.^1^ The fracture mode fits well with the 3‐point bending results, as all groups exhibit/share a completely cohesive fracture mode. This means that the metal*–*ceramic bond strength is higher than the cohesive strength of bonding agent/opaque, which is of course the same for all groups. Therefore, an increase in porcelain‐fused‐to‐metal bonding requires the development of bonding agents with higher cohesive strength.

The specimens for interfacial analysis were prepared by omitting the stage of surface cleaning with airborne Al_2_O_3_ particles contrary to the manufacturer's instructions. This stage is omitted to facilitate the discrimination of the porcelain and alloy at high magnifications^13^, ^35^ and to avoid the contribution of aluminum (Al) and O from the retained alumina fragments^36^ to the elemental distribution of the aforementioned elements.

All groups demonstrated a continuous interface with porcelain without pores, debonding areas, or other defects, implying that the bonding agent spread efficiently even on a polished metallic substrate, which is in accordance with previous studies.^13^ This is also achieved by an opaque layer without the use of the bonding agent.^25^, ^31^ Line scan analysis did not show any local peaks of probed elements at the porcelain metal interfaces (Figure 4), a finding in agreement with previous reports on this bonding agent.^13^ Among the four major elements found at the interface, Co showed the highest diffusion rate in all cases, while Ti had the lowest diffusion rate. In a previous study, a thermodynamic approach based on the chemical affinity of these elements with O based on Ellingham's diagram was presented.^13^ Among Co, Cr, and Ti, the latter has a higher chemical affinity for O, implying that it has a higher tendency to react with this specific element. Even if Co and Cr react first with O during the formation of surface oxides or for kinetic reasons that dominate the reaction rate of each element at the interface, Ti can still reduce metal oxides through metallothermic reduction.^37^ This means that elements with higher chemical affinity with O (*M*_HA_) will reduce metallic oxides of metals with lower chemical affinity (*M*_LA_) according to the lower reaction:

(3) $$M_{\mathrm{LA}}O+M_{\mathrm{HA}}\to M_{\mathrm{HA}}+M_{\mathrm{LA}}O$$

Co and Cr, which dominate the native metal oxide at the interface, are released. Therefore, Co and Cr as free ions can diffuse further from the surface oxide region, while Ti keeps moving toward the metallic surface through metallothermic reduction. Despite the agreement between the thermodynamic data and the experimental results, it should be mentioned that Ellingham diagrams have been calculated based on the reaction of pure elements with *O* and thus should be recalculated when these elements are not in a pure state, which is a limitation of this approach, as these thermodynamic data are not currently available.

Although analytical techniques with high resolution (i.e., Transmission Electron Microscopy) are required for more accurate interfacial characterization of this multielement and multicompound joint, some researchers use this diffusion zone for a rough estimate of the extent of the interface.^25^, ^31^ The interfacial layer was estimated to range from 12∼13 μm for a cast group^25^ and 8∼9 μm^25^ and 7∼10 μm^31^ for an SLM group, which is profoundly different by approximately 3.2 μm if the largest value of Table 4 is considered. These differences should be appended either to the use of the bonding agent or the polishing of the surface and omission of the sandblasting step for the reasons explained above. In general, if the oxide layer is absent, as in the case of gold electroformed crowns (galvanoceramic),^38^ or excessively thick, as in the case of Ti, ^35^ then poor bond strengths ensue.^16^, ^39^ The intermediate values of the interfacial zone obtained in this study support high bond strengths, while a similar extent of the interfacial layer fits with the absence of significant differences in bond strength among groups.

From a summary of the results of his study and the data from the literature, it seems that the bond strength is independent of the CoCr alloy type or the manufacturing process when conventional casting and SLM are considered, while the selection of dental porcelain seems to play a decisive role in this property.

## CONCLUSIONS

Under the limitations of this research study, the findings indicate that metal‐ceramic bond strength is independent of contemporary CoCr alloy types and manufacturing processes if conventional casting and SLM are compared. Interfacial analysis did not provide any difference among the groups tested.

## CONFLICT OF INTEREST STATEMENT

None to declare.
